# LncRNA *AC142119.1* facilitates the progression of neuroblastoma by epigenetically initiating the transcription of *MYCN*

**DOI:** 10.1186/s12967-023-04535-3

**Published:** 2023-09-23

**Authors:** Rui Yang, Nanjing Liu, Ting Li, Fangjie Liu, Jun Zhang, Hui Zhao, Lin Zou, Xiaoyan He

**Affiliations:** 1 Center for Clinical Molecular Medicine, National Clinical Research Center for Child Health and Disorders, Ministry of Education Key Laboratory of Child Development and Disorders, China International Science and Technology Cooperation Base of Child Development and Critical Disorders, Chongqing Key Laboratory of Pediatrics, Children’s Hospital of Chongqing Medical University, Chongqing, 400014 China; 2 Chongqing Key Laboratory of Translational Research for Cancer Metastasis and Individualized Treatment, Chongqing University Cancer Hospital, Chongqing, 400030 China; 3 Key Laboratory of Laboratory Medical Diagnostics, Ministry of Education, Chongqing Medical University, Chongqing, 400016 China; 4 Department of Oncological Surgery, Children’s Hospital of Chongqing Medical University, Chongqing, 400014 China; 5 School of Biomedical Sciences, Faculty of Medicine, The Chinese University of Hong Kong, Hong Kong, 999077 China; 6 Clinical Research Unit, Children’s Hospital of Shanghai Jiaotong University School of Medicine, Institute of Pediatric Infection, Immunity, and Critical Care Medicine, Shanghai Jiaotong University School of Medicine, Shanghai, 200062 China

**Keywords:** Neuroblastoma, lncRNA *AC142119.1*, *MYCN*, WDR5

## Abstract

**Background:**

Oncogene *MYCN* is closely related with malignant progression and poor prognosis of neuroblastoma (NB). Recently, long non-coding RNAs (lncRNAs) have been recognized as crucial regulators in various cancers. However, whether lncRNAs contribute to the overexpression of *MYCN* in NB is unclear.

**Methods:**

Microarray analysis were applied to analyze the differentially expressed lncRNAs between *MYCN*-amplified and *MYCN*-non-amplified NB cell lines. Bioinformatic analyses were utilized to identify lncRNAs nearby *MYCN* locus. qRT-PCR was used to detect the expression level of lncRNA *AC142119.1* in NB cell lines and tissues. Gain- and loss-of-function assays were conducted to investigate the biological effect of *AC142119.1* in NB. Fluorescence in situ hybridization, RNA pull-down, RNA immunoprecipitation, mass spectrometry, RNA electrophoretic mobility shift, chromatin immunoprecipitation and chromatin isolation by RNA purification assays were performed to validate the interaction between *AC142119.1* and WDR5 protein as well as *MYCN* promoter*.*

**Results:**

*AC142119.1* was significantly elevated in NB tissues with *MYCN* amplification, advanced INSS stage and high risk, and associated with poor survival of NB patients. Moreover, enforced expression of *AC142119.1* reinforced the proliferation of NB cells in vitro and in vivo. Additionally, *AC142119.1* specifically recruited WDR5 protein to interact with *MYCN* promoter, further initiating the transcription of *MYCN* and accelerating NB progression.

**Conclusions:**

We identified a novel lncRNA *AC142119.1*, which promoted the progression of NB through epigenetically initiating the transcription of *MYCN* via interacting with both WDR5 protein and the promoter of *MYCN*, indicating that *AC142119.1* might be a potential diagnostic biomarker and therapeutic target for NB.

**Supplementary Information:**

The online version contains supplementary material available at 10.1186/s12967-023-04535-3.

## Background

Neuroblastoma (NB) is the most prevalent extra-cranial solid tumor in young children, which is a malignancy of the sympathetic nervous system, arising from the neural crest during the embryonic development [1, 2]. It accounts for about 6–10% of all childhood tumors but 12–15% of all cancer deaths in children [3]. NB has been characterized with a high heterogeneity in clinical presentations, ranging from spontaneous regression tumors without any medical intervention to highly aggressive and metastatic diseases that are resistant to multimodal anti-cancer therapies [4, 5]. This heterogeneity leads to great differences in the survival of NB patients. The survival rate of patients diagnosed with low- and intermediate‐risk NB is greater than 90%, whereas the 5-year survival rate of the patients with high-risk NB is less than 50% [6, 7]. Due to the insidious symptoms, the majority of patients were diagnosed with high-risk NB, it is necessary to identify novel therapeutic targets to improve the prognosis of NB patients.

*MYCN* belongs to the *MYC* proto-oncogene family and encodes a transcription factor that modulates the expression of numerous target genes, which engage in a broad spectrum of intracellular biological process including cell proliferation, apoptosis, senescence, differentiation, metabolism, DNA damage repair and protein synthesis [8, 9]. Amplification of the *MYCN* gene has been one of the earliest genetic markers in NB and occurs in approximately 22% of all NB and 45% of high-risk NB cases [10, 11]. *MYCN* gene amplification and consequent *MYCN* oncoprotein overexpression are strongly correlated with particularly poor prognosis of NB patients, and defines an aggressive subset of NB [11]. Laboratory evidences also indicated that exogenous expression of *MYCN* in NB cells with non-amplified *MYCN* enhanced the cell proliferation and tumorigenic capacity [12]. Additional studies further suggested that ectopic expression of *MYCN* is sufficient to drive *MYCN*-amplified NB in mice and zebrafish models [13, 14]. However, the underlying mechanisms of *MYCN* overexpression in NB are not fully illustrated up to date.

Long non-coding RNAs (lncRNAs) are a class of transcripts with a length of more than 200 nucleotides that lack the ability to encode proteins [15]. Recent years, lncRNAs have been proposed to serve as key regulators encompassed complex functions in various human cancers, including NB, and have attracted increasing attention [16]. Previous studies demonstrated that lncRNAs could regulate epigenetic modification, gene transcription, translation, the stability of RNA/protein and post-translational modification via interacting with DNA, RNAs and/or proteins, thus affecting the biological behaviors of cancer cells [17–19]. In NB, it has been demonstrated that lncRNA *HNF4A-AS1* enhanced the aerobic glycolysis and growth of NB cells by transactivation of CTCF and transcriptional alteration of *HNF4A* via binding with hnRNPU [20]. Moreover, lncRNA *NHEG1* physically interacted with and stabilized DDX5 protein, resulting in the transactivation of *β-catenin* and progression of NB [21]. However, only a few such lncRNAs have been well-characterized in NB, the biological function and underlying mechanisms of the great majority of these transcripts in the initiation and progression of NB remain unexplored.

Here, we identified a novel *MYCN*-related lncRNA *AC142119.1*, which was significantly upregulated in *MYCN*-amplified NB tissues and cell lines, compared with those without *MYCN* amplification, and high expression of *AC142119.1* was positively associated with poor survival in NB patients. Furthermore, we uncovered that *AC142119.1* could recruit WDR5 protein to interact with the promoter of *MYCN*, further activate the transcription of *MYCN* and eventually accelerate the progression of NB. The data present here provided novel insights into the potential mechanism by which *AC142119.1* contributed to the overexpression of *MYCN* in NB, implying that *AC142119.1* might be a promising therapeutic target for NB.

## Methods

### Cell culture

Human neuroblastoma cell lines (SH-SY5Y, SK-N-AS, IMR-32 and SK-N-DZ) were purchased from American Type Culture Collection (ATCC, Manassas, VA, USA). All these cells were maintained in DMEM medium (Gibco, Carlsbad, CA, USA) supplemented with 10% fetal bovine serum, 100 U/ml penicillin and 100 μg/ml streptomycin. Cells were cultured at 37 ℃ with 5% CO_2_ in a humidified atmosphere.

### Clinical samples

The 55 cases of NB tissue specimens were collected from the randomly selected patients who were diagnosed with NB at Children’s Hospital of Chongqing Medical University (Chongqing, China) in compliance with routine clinical sampling after obtaining the written informed consent from their patient or legal guardian. None of these patients received chemotherapy or radiotherapy prior to surgical resection. The present study conformed to the Declaration of Helsinki and was approved by the Ethics Committee of Children’s Hospital of Chongqing Medical University (Chongqing, China).

### RNA isolation, microarray analysis

Total RNA of NB cells and tissues were extracted with TRIzol reagent (Invitrogen, Carlsbad, CA, USA) following the manufacturer’s instructions. The purity and quantity of the isolated RNA were evaluated by Nanodrop ND-1000 spectrophotometer (Thermo Fisher Scientific, Waltham, MA, USA). The RNA integrity was examined by denaturing agarose gel electrophoresis. The Arraystar Human LncRNA microarray V3.0 (Arraystar, USA) was applied to obtain the lncRNA expression profile. The sample labeling, microarray hybridization and data analysis were conducted by Kangcheng Bio-tech (Shanghai, China).

### Rapid amplification of cDNA ends (RACE) analysis

RACE assay was performed with GeneRacer^™^ Kit (Invitrogen) in accordance with the manufacturer’s instructions to determine the full length of *AC142119.1* transcript in SK-N-DZ cells. The products of RACE PCR were resolved on 1.5% agarose gel. The gel products were purified using SanPrep Column DNA Gel Extraction Kit (Sangon Biotech, Shanghai, China) and cloned into pGM-T vector for sequencing. The specific primers were listed in Additional file 1: Table S1.

### qRT-PCR

Total RNA was reverse transcribed into cDNA with PrimeScript RT-PCR Kit (Takara, Dalian, China) based on the manufacturer’s protocols. Then the cDNA was amplified with SYBR Green Premix Ex Taq (Takara). The relative expression of the target genes was assessed by 2^–ΔΔCT^ method, with GAPDH serving as an internal reference. The primer sequences were displayed in Additional file 1: Table S1.

### Vectors, GapmeRs, siRNAs and cell transfection

The full-length cDNA of *AC142119.1* was synthesized and inserted into the lentiviral vector pHBLV-CMVIE-ZsGreen-T2A-puro to establish *AC142119.1* overexpression vector, with the empty vector without *AC142119.1* sequence serving as a control. To knockdown *AC142119.1*, LNA-modified antisense oligonucleotides (GapmeRs) targeting *AC142119.1* and negative control were synthesized by QIAGEN (Hilden, Germany). Full-length cDNA of human *WDR5* and its truncations were amplified and subcloned into pcDNA3.1 vector. The full-length cDNA of human *MYCN* was inserted into pcDNA3.1 vector to establish *MYCN* overexpression vector. Specific siRNAs targeting *MYCN* or *WDR5* and negative control were purchased from Sangon Biotech. Cell transfection was performed with Lipofectamine 2000 (Invitrogen) in line with the manufacturer’s instructions. The sequences of the GapmeRs and siRNAs were presented in Additional file 1: Table S2.

### Lentivirus packaging and infection

To generate lentivirus, the lentiviral vectors were co-transfected with packaging plasmids (pMD2G and psPAX2) into HEK293T cells using Lipofectamine 2000 (Invitrogen). The supernatants containing lentiviral particles were harvested at 48 h and 72 h after transfection, which were then concentrated by ultracentrifugation at 120,000 *g* for 2 h. The NB cells were directly infected with the obtained lentivirus, followed by selecting with puromycin 48 h after infection to produce stable cells.

### Protein extraction and western blot assay

Total proteins of NB cells were extracted using RIPA buffer (Beyotime, Shanghai, China) supplemented with 1% PMSF. The proteins were quantified by Enhanced BCA Protein Assay Kit (Beyotime), separated by 10% SDS-PAGE and transferred onto PVDF membranes (Millipore, Billerica, MA, USA). After blocked with 5% skimmed milk, the bands were incubated with primary antibodies against MYCN (1:1000, CST, Beverly, MA, USA) and GAPDH (1:3000, CST) at 4 ℃ overnight, and then incubated with HRP-conjugated secondary antibodies (1:3000, CST) at room temperature for 2 h. Finally, the bands were visualized with enhanced chemiluminescence system.

### Cytoplasmic and nuclear fractionation

The cytoplasmic and nuclear RNA of NB cells were isolated according to the recommended protocols of PARIS^™^ Kit (Thermo Fisher Scientific). Briefly, the cells were lysed with cell fractionation buffer, followed by low-speed centrifugation to separate the nuclear fraction from the cytoplasmic fraction. The supernatant containing the cytoplasmic fraction was carefully collected and the nuclear pellet was added with cell disruption buffer. Then, the split fractions were mixed with 2 × lysis/binding solution and 100% ethanol and treated with filtration columns. After that, the samples were washed with wash solution and eluted with elution solution.

### Fluorescence in situ hybridization (FISH)

FISH assay was performed with Cy3-labeled probes targeting *AC142119.1* and Fluorescent In Situ Hybridization kit (RiboBio) following the manufacturer’s instructions. Briefly, the NB cells seeded on the coverslips were fixed with 4% paraformaldehyde and permeabilized with 0.5% Triton X-100. After incubating with prehybridization buffer at 37 ℃ for 30 min, the cells were hybridized with hybridization buffer containing specific probes at 37 ℃ overnight. Then, cover slips were washed with 2 × SSC for 6 times and followed by counterstaining with DAPI.

### Cell proliferation and cell cycle assays

The proliferation of NB cells was determined by the growth curves generated from Cell Counting Kit-8 (Beyotime) in accordance with the manufacturer’s protocols. For cell cycle analysis, NB cells were collected and treated with 75% ice-cold ethanol. Then, the cells were stained with PI at room temperature for 30 min and analyzed by a flow cytometer.

### Xenograft model

The four-week-old BALB/c nude mice were purchased from the Center of Experimental Animals of Chongqing Medical University (Chongqing, China) and fed under the standard conditions. Stable SK-N-AS cells (5 × 10^6^) were subcutaneously injected into the dorsal flanks of the randomly grouped nude mice to construct *AC142119.1* overexpression xenograft models. As to *AC142119.1* knockdown models, SK-N-DZ cells (5 × 10^6^) were inoculated into the mice. When the xenograft tumor grew to 1 mm^3^, LNA-modified GapmeR targeting *AC142119.1* transcript was injected into the tumor of experiment group once every 3 days. The volumes of the tumors were monitored once a week and calculated with the formula, 0.5 × width^2^ × length. Four weeks later, the tumor-bearing mice were sacrificed and followed by tumor resection and weighing. The animal studies were approved by Chongqing Medical University Animal Care and Use Committee.

### Immunohistochemistry (IHC)

For IHC staining, after dewaxed and rehydrated, the paraffin-embedded tissue sections were incubated with primary antibodies against MYCN (1:200, CST) or Ki67 (1:500, CST) at 4 ℃ overnight and biotin-labeled secondary antibodies at 37 ℃ for 1 h. The sections were then stained with DAB and hematoxylin.

### RNA immunoprecipitation (RIP)

RIP assay was executed with antibody against WDR5 (CST), IgG control and a Magna RIP RNA Binding Protein Immunoprecipitation Kit (Millipore) following the manufacturer’s instruction.

### RNA pull-down assay and mass spectrometry

The biotin-labeled full-length and truncated *AC142119.1* RNA were transcribed in vitro using T7 RNA polymerase with PCR products used as templates and labeled with RNA 3′ End Desthiobiotinylation Kit (Thermo Fisher Scientific) in accordance with the recommended conditions. RNA pull-down assays were conducted using Pierce™ Magnetic RNA–Protein Pull-Down Kit (Thermo Fisher Scientific) according to the standard protocols. In brief, the NB cells were lysed with RIP lysis buffer supplemented with Protease Inhibitor Cocktail (Thermo Fisher Scientific) and RNase inhibitors, and then incubated with biotin-labeled RNAs combined with streptavidin magnetic beads at 4 ℃ for 1 h. The RNA-binding protein complexes were collected with a magnetic stand and eluted in elution buffer after washing 3 times with wash buffer. The pulled down proteins were analyzed by mass spectrometry at Sangon Biotech or western blot.

### RNA electrophoretic mobility shift assay (EMSA)

The biotin-labeled probe specific for *AC142119.1* transcript was synthesized by Sangon Biotech. RNA EMSAs were carried out using LightShift^®^ Chemiluminescent RNA EMSA Kit (Thermo Fisher Scientific) following the manufacturer’s instructions. Briefly, the nuclear extracts of NB cells transfected with WDR5 expression vector or its deletion mutations together with biotin-tagged *AC142119.1* probe were incubated in the binding reactions at room temperature for 30 min. Then, the reactions were added with loading buffer and subjected to PAGE using native gels, followed by transferring onto nylon membranes. After crosslinking to the membranes by exposing to UV irradiation, the biotin-labeled probe was detected by streptavidin-HRP conjugate and enhanced chemiluminescence.

### Chromatin immunoprecipitation (ChIP) assay

ChIP assays were conducted using SimpleChIP^®^ Enzymatic Chromatin IP Kit (CST) with antibodies specific for H3K4me3 (CST) or IgG control. The precipitated DNA fragments were subjected to qRT-PCR analysis.

### Chromatin isolation by RNA purification (ChIRP) assay

The biotin-labeled probes targeting *AC142119.1* transcript and negative control probes were synthesized by Sangon Biotech. The NB cells were fixed with 1% formaldehyde at room temperature for 10 min and terminated with 2.5 M glycine for 5 min. Then, the cells were lysed and sonicated to shear the chromatin into 100–200 bp fragments. After centrifugation at 12,000 *g* for 10 min, the supernatant of the sonicated samples was collected and added with 2 × lysate volume of DNA-RNA hybridization buffer, followed by incubating with biotin-labeled probes at room temperature with gentle shaking overnight. Next, the pre-washed streptavidin beads were added into each of the hybridization reaction and incubated at room temperature for 4 h. After the step of 5 times washing, the beads were collected with a magnetic stand and eluted in elution buffer. Finally, the bound RNA and DNA fragments were purified from the beads.

### Statistical analysis

All the statistical analyses were conducted with SPSS 21.0. Differences between two or more groups were assessed by student’s t-test or one-way ANOVA, respectively. The relevance between two groups were determined by Pearson correlation. *P* < 0.05 was considered as statistically significant.

## Results

### Identification of the MYCN-related lncRNA AC142119.1

**Fig.1:**
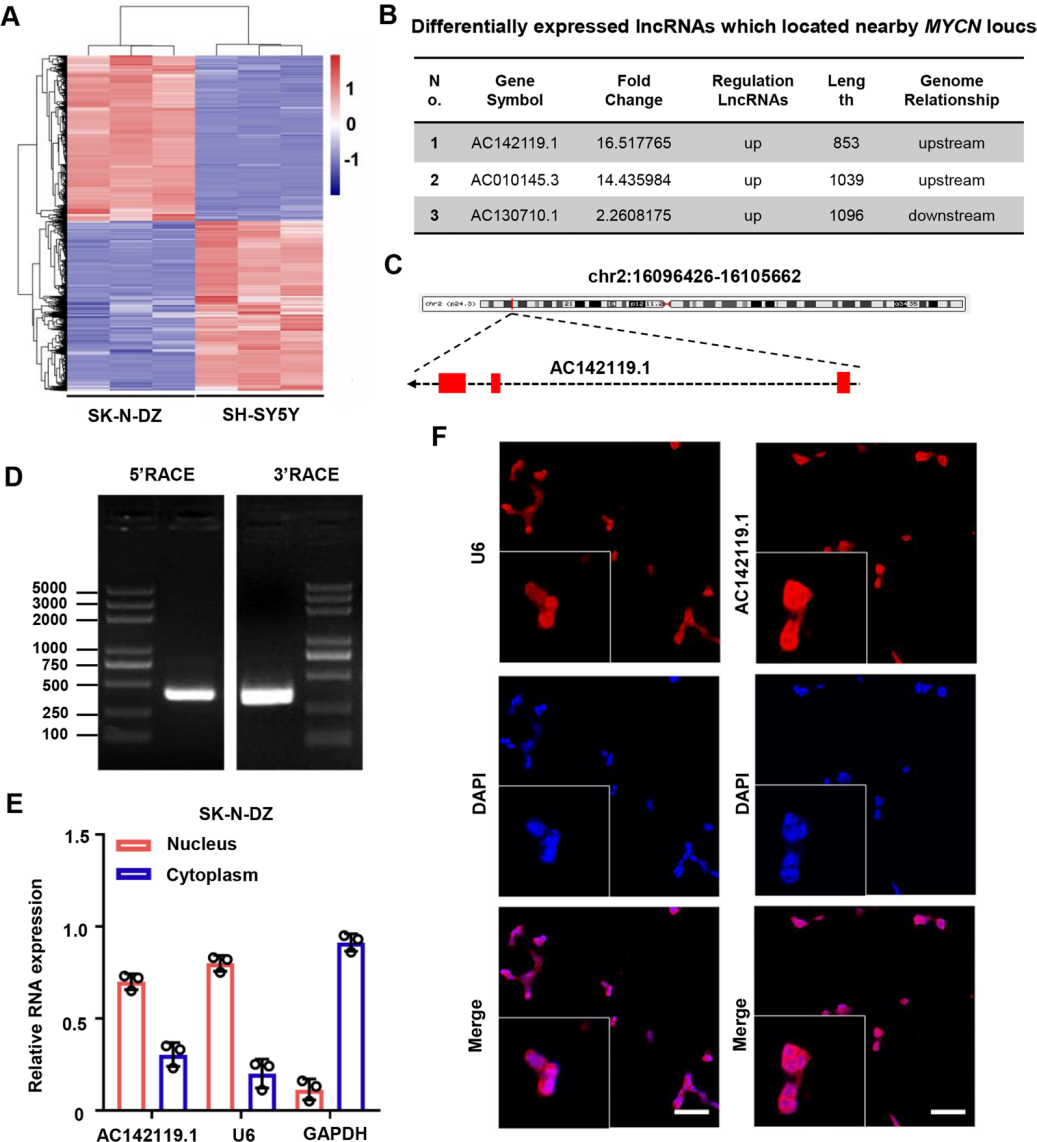
Identification of the *MYCN*-related lncRNA *AC142119.1*. **A** Heatmap depicts the differentially expressed lncRNAs between *MYCN*-amplified cell line SK-N-DZ and *MYCN*-non-amplified cell line SH-SY5Y. **B** Three lncRNAs candidates located nearby the *MYCN* locus. **C** The genomic location of *AC142119.1* annotated by UCSC database. **D** PCR products of 5′ and 3′ RACE analyses subjected to agarose gel electrophoresis. **E** Cytoplasmic and nuclear fractionation of SK-N-DZ cells followed by qRT-PCR to determine the subcellular localization of *AC142119.1* RNA. *GAPDH* and *U6* were used as cytoplasmic and nuclear marker, respectively. **F** FISH detection of *AC142119.1* transcript in SK-N-DZ cells. Scale bar, 50 μm

In order to identify the lncRNAs that are important for *MYCN* amplification-induced tumorigenesis of NB, we analyzed the lncRNA expression profile using microarray in *MYCN*-amplified cell line SK-N-DZ and *MYCN*-non-amplified cell line SH-SY5Y. Differential expression analysis indicated that 1608 lncRNAs were upregulated and 1647 lncRNAs were downregulated in *MYCN*-amplified SK-N-DZ cells with the cut-off criteria of fold change > 2 and *P* < 0.05 (Fig. 1A), including *lncNB1* (also known as *RP1-40E16.9*), *AF127936.7* and *RP11-102F4.3* that have been previously reported to be significantly increased in *MYCN*-amplified NB cell lines, which proved the robustness of our data to a certain extent [22]. Considering that lncRNAs are known to modulate their neighboring protein-coding genes *in cis*. We identified three lncRNA candidates (*AC142119.1*, *AC010145.3* and *AC130710.1*) of these differentially expressed lncRNAs that were located nearby the *MYCN* locus. To prioritize the most *MYCN*-relevant and biologically significant lncRNA, we concentrated on *AC142119.1*, as it was the most upregulated one (16.52 folds) in *MYCN*-amplified SK-N-DZ cell line and resided upstream of *MYCN* locus (Fig. 1B, C). According to the annotation of UCSC database (http://genome.ucsc.edu/), *AC142119.1* was a lncRNA containing 3 exons located at chr2: 16096426-16105662 and predicted to produce two transcripts with length of 853nt and 642nt, respectively. To confirm the full-length transcripts of *AC142119.1* in NB cells, both 5′ RACE and 3′ RACE assays were executed and determined a novel isoform of *AC142119.1* with a length of 924nt in SK-N-DZ cells (Fig. 1D and Additional file 2: Fig. S1A), which was discordant with the prediction of UCSC database. Subsequent analyses of the novel *AC142119.1* transcript using PRIDE, PhyloCSF, CPAT, Bazzini small ORFs and Lee translation initiation sites revealed the low coding potential of *AC142119.1*, corroborating the identity of *AC142119.1* as a non-coding gene (Additional file 2: Fig. S1B). Cellular fractionation assays followed by qRT-PCR analysis disclosed that *AC142119.1* was predominately localized in the nucleus of SK-N-DZ cells with a small proportion distributing in the cytoplasm (Fig. 1E), which was further substantiated by FISH assay with specific probes (Fig. 1F). All the above data suggested that lncRNA *AC142119.1* was a novel non-coding gene and highly expressed in *MYCN*-amplified NB cells.

### *AC142119.1* is elevated in *MYCN*-amplified NB tissues and positively correlated with the poor outcome in NB patients

**Fig. 2:**
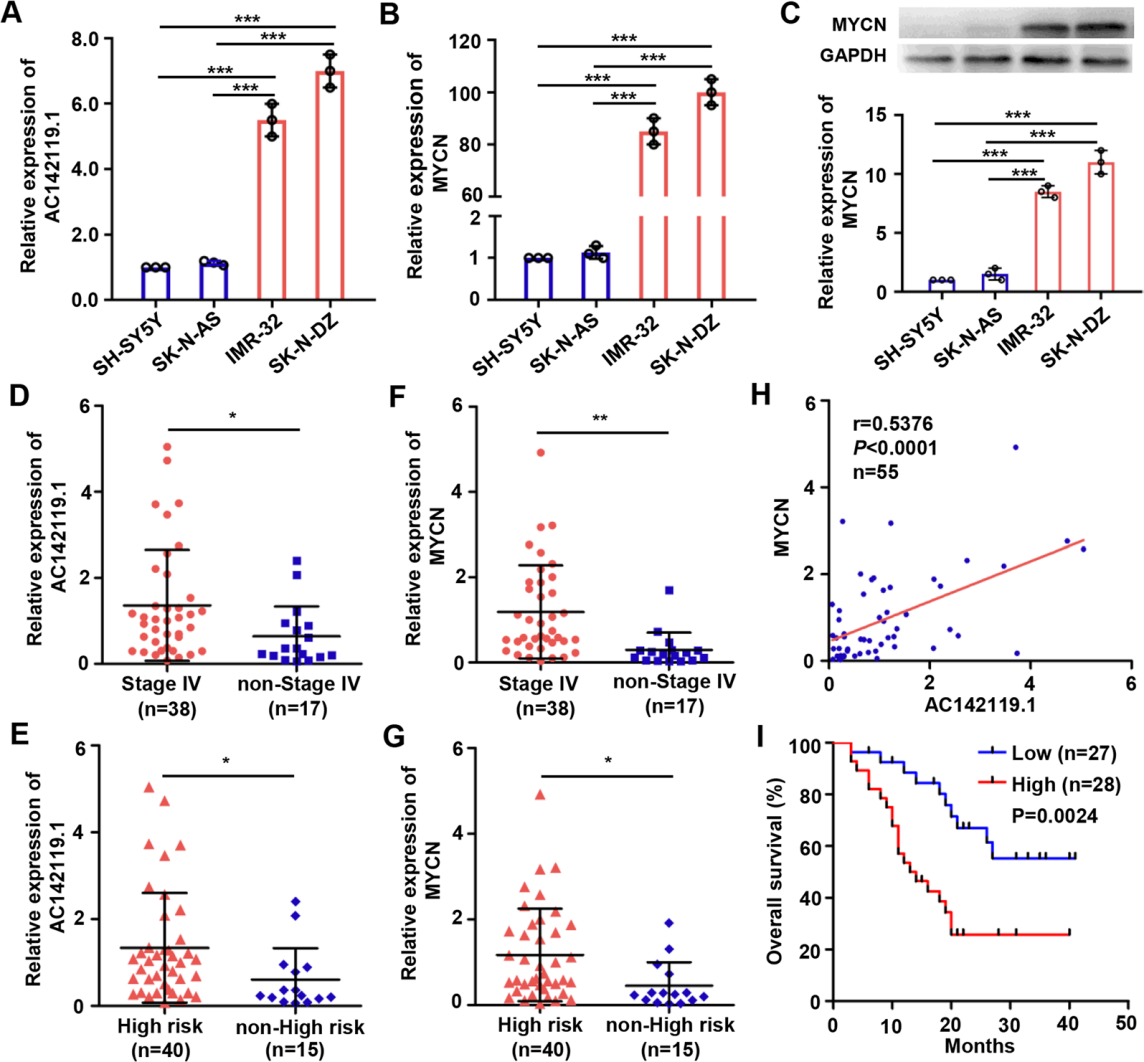
*AC142119.1* is elevated in *MYCN*-amplified NB tissues and positively correlated with the poor survival in NB patients.** A** qRT-PCR to analyze the expression of *AC142119.1* in *MYCN*-amplified and non-amplified NB cell lines.** B**, **C** The mRNA (**B**) and protein (**C**) levels of *MYCN* in *MYCN*-amplified and non-amplified NB cell lines were determined by qRT-PCR and western blot, respectively. **D**, **F** Relative expression of *AC142119.1* (**D**) and *MYCN* (**F**) in NB patients with or without stage IV. **E**, **G** Relative expression of *AC142119.1 *(**E**) and *MYCN* (**G**) in NB patients with or without high-risk. **H** Pearson correlation analysis between the expression of *AC142119.1* and *MYCN* in 55 NB tissues. **I** The survival probability of NB patients was analyzed by Kaplan–Meier method. The patients were categorized into low and high group according the median of *AC142119.1* expression in all NB patients. *P < 0.05, **P < 0.01, ***P < 0.001

In order to determine the expression pattern of *AC142119.1* in NB, qRT-PCR was performed in two *MYCN*-amplified (SK-N-DZ and IMR-32 cells) and non-amplified (SH-SY5Y and SK-N-AS cells) NB cell lines. As a result, cells with *MYCN* amplification showed considerably higher expression levels of *AC142119.1* than those without *MYCN* amplification (Fig. 2A). Meanwhile, *MYCN*-amplified NB cell lines also expressed high levels of *MYCN* mRNA and protein compared with *MYCN*-non-amplified cell lines (Fig. 2B, C). Next, we examined the expression levels of *AC142119.1* in randomly collected 55 NB tissues, finding that NB patients with advanced INSS stage and high risk showed much higher expression levels of *AC142119.1* than those in control groups (Fig. 2D, E). Consistently, *MYCN* exhibited analogous expression patterns with *AC142119.1* in NB tissues (Fig. 2F, G). Moreover, Pearson correlation analysis revealed a strong positive correlation between *AC142119.1* and *MYCN* mRNA levels in these NB tissues (Fig. 2H). Importantly, Kaplan–Meier survival curve showed that the expression level of *AC142119.1* was negatively correlated with the overall survival of NB patients (Fig. 2I). These findings established that *AC142119.1* was positively correlated with *MYCN* and may contribute to the progression of NB.

### *AC142119.1* facilitates the proliferation of NB cells in vitro

**Fig. 3:**
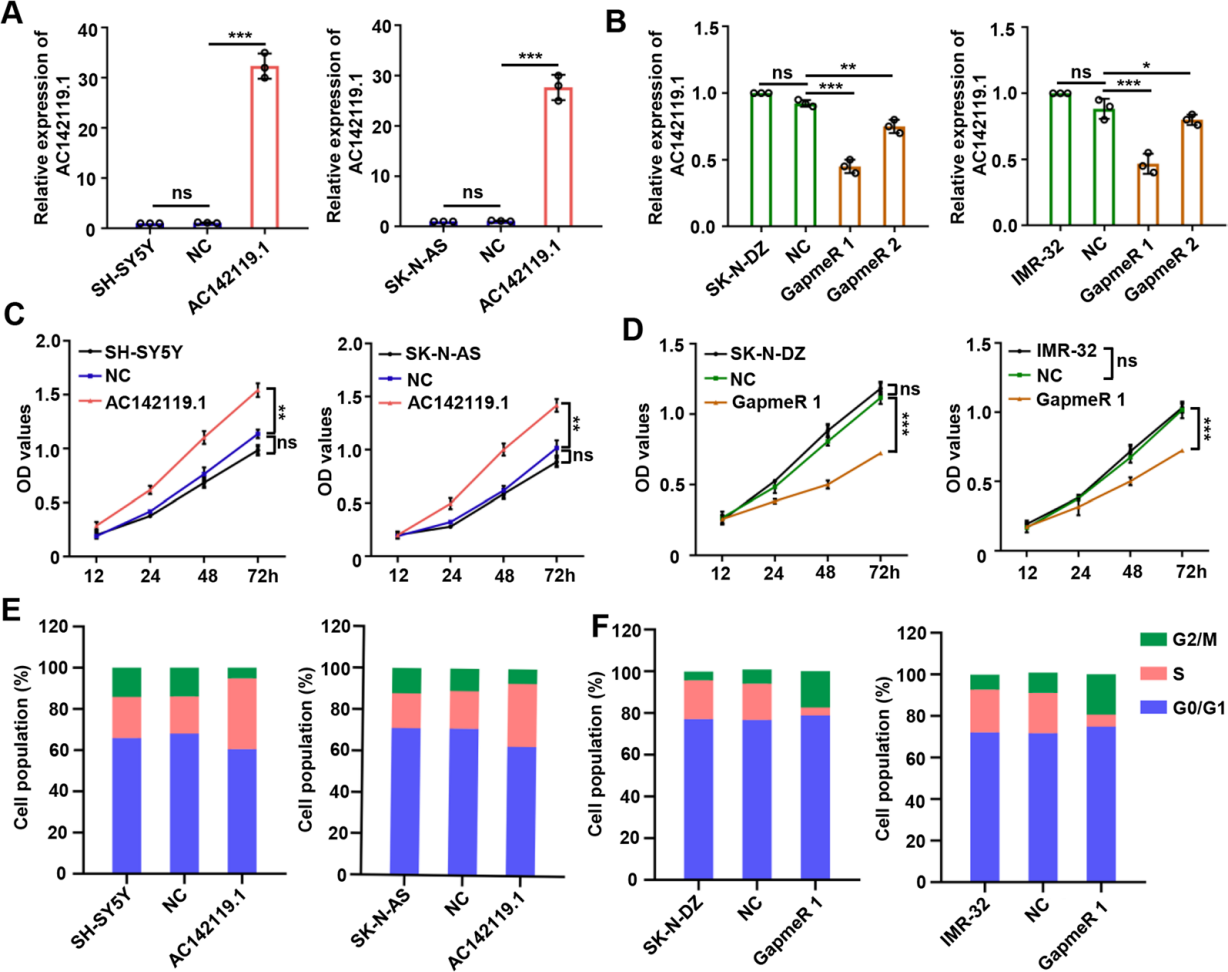
*AC142119.1* facilitates the proliferation of NB cells in vitro.** A**, **B** The expression of *AC142119.1* in NB cell lines after overexpression (**A**) or knockdown (**B**) of *AC142119.1* was assessed by qRT-PCR. **C**, **D** Growth curves generated from CCK-8 assays to determine the proliferation of NB cell lines transfected with indicated vectors (**C**) or GapmeRs (**D**). **E**, **F** Cell cycle progression of NB cell lines were analyzed by flow cytometry after enforced expression (**E**) or silencing (**F**) of *AC142119.1*. *P < 0.05, **P < 0.01, ***P < 0.001, ns, no significance

To investigate the biological role of *AC142119.1* in NB, we stably overexpressed *AC142119.1* in SK-N-AS and SH-SY5Y, two cell lines with low level of *AC142119.1*, by infecting with lentivirus carrying *AC142119.1*, which was confirmed by qRT-PCR (Fig. 3A). Given that *AC142119.1* was localized in the nuclear of NB cells, LNA-modified antisense oligonucleotides (GapmeRs) were applied in SK-N-DZ and IMR-32 cells with high level of *AC142119.1* to knock down *AC142119.1* (Fig. 3B). Then, we detected the viability of the NB cells using CCK-8 assays, finding that enforced expression of *AC142119.1* enhanced the proliferation of SK-N-AS and SH-SY5Y cells (Fig. 3C), whereas silencing of *AC142119.1* inhibited the growth of SK-N-DZ and IMR-32 cells (Fig. 3D). In addition, we also assessed whether *AC142119.1* affects the cell cycle of NB cells. As anticipated, SK-N-AS and SH-SY5Y cells with overexpressed *AC142119.1* had larger proportion of S phase and smaller proportion of G1 phase than those in control group by flow cytometry analyses (Fig. 3E), while deficiency of *AC142119.1* produced an entirely opposite effect in SK-N-DZ and IMR-32 cells, implying the proliferation-promoting effect of *AC142119.1* on NB cells (Fig. 3F). Collectively, these results substantiated that *AC142119.1* acted as an oncogenic driver in NB.

### *AC142119.1* contributes to the tumorigenesis of NB in vivo

**Fig. 4:**
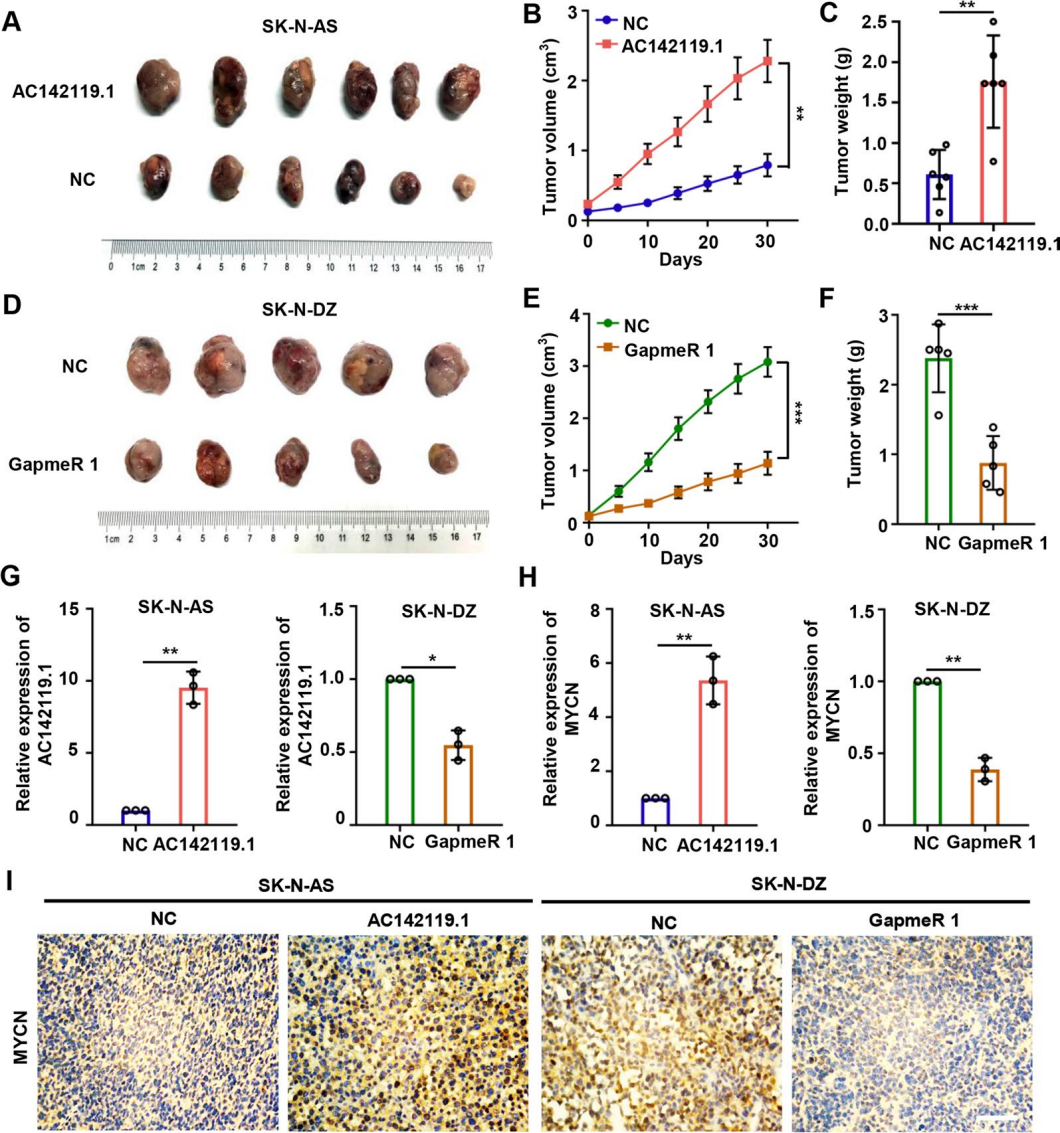
*AC142119.1* contributes to the tumorigenesis and progression of NB in vivo.** A**, **D** Images of xenograft tumors derived from the *AC142119.1* overexpression (**A**) or knockdown (**D**) models. **B**, **E** Tumor volume was measured once a week. **C**, **F** The tumors were weighed after resection. **G**, **H** The expression of *AC142119.1* (**G**) and *MYCN* (**H**) in xenograft tumors was evaluated by qRT-PCR. **I** IHC staining to examine the protein level of *MYCN* in xenograft tumors. Scale bar, 100 μm. *P < 0.05, **P < 0.01, ***P < 0.001

To further probe the effect of *AC142119.1* on tumorigenesis of NB in vivo, SK-N-AS cells with stable overexpression of *AC142119.1* and control cells were subcutaneously inoculated into the dorsal flank of nude mice to construct xenograft models. We observed that enforced expression of *AC142119.1* significantly accelerated the formation and growth of xenograft tumors (Fig. 4A–C). Furthermore, we also established *AC142119.1* knockdown xenograft models by inoculating the mice with SK-N-DZ cells and treating the tumors with LNA-modified GapmeR. Four weeks later, tumors treated with LNA-modified GapmeR specific for *AC142119.1* had much smaller volumes and lower weights than those in control group, pinpointing that depletion of *AC142119.1* was unfavorable for the growth of xenograft tumors (Fig. 4D–F). To validate the regulatory role of *AC142119.1* on *MYCN *in vivo, we analyzed the expression of *MYCN* in these xenograft tumors. qRT-PCR analyses uncovered that the mRNA level of *MYCN* were significantly increased in *AC142119.1* overexpression group while decreased in the *AC142119.1* knockdown group (Fig. 4G, H). Similarly, IHC staining indicated that tumors with overexpressed *AC142119.1* had higher MYCN and Ki67 protein levels than control group, whereas downregulation of *AC142119.1* generated a reverse effect (Fig. 4I and Additional file 3: Fig. S2). These data demonstrated that *AC142119.1* facilitates the tumorigenesis of NB in vivo.

### *AC142119.1* directly binds with WDR5 in NB cells

**Fig. 5:**
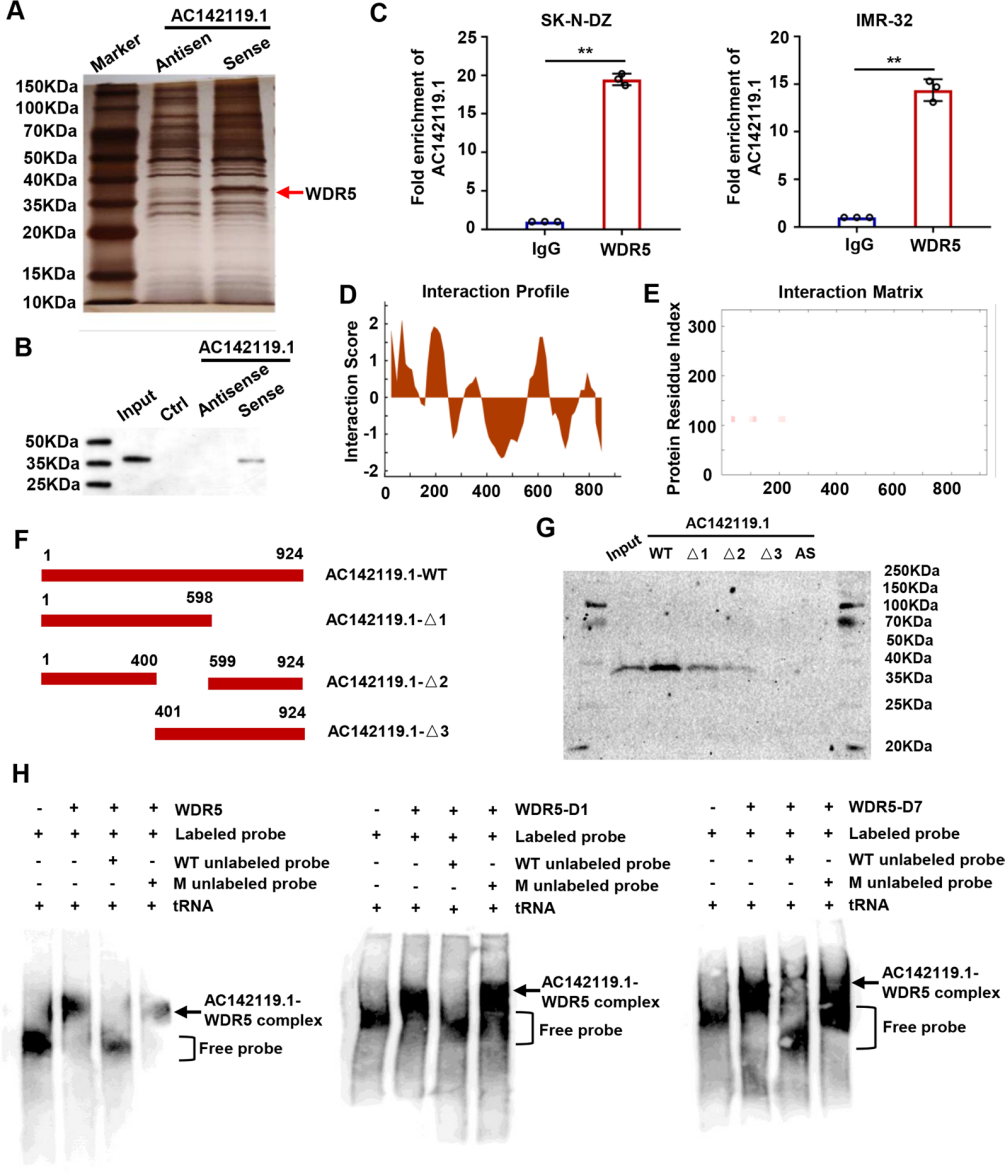
*AC142119.1* directly binds with WDR5 in NB cells.** A** Silver staining of the SDS-PAGE gel showing the separated proteins pulled down by biotin-labeled *AC142119.1* RNA. **B** Western blot analysis of WDR5 in protein samples captured by RNA pull-down assay. **C** RIP assays to validate the interaction between endogenous *AC142119.1* transcript and WDR5 in SK-N-DZ and IMR-32 cells. **D**, **E** Interaction profile (**D**) and matrix (**E**) between *AC142119.1* transcript and WDR5 protein predicted by catRAPID database. **F** Schematic diagram of full-length and truncated *AC142119.1* RNA. **G** RNA pull-down assays were conducted with biotin labeled full-length and truncated *AC142119.1* transcript. The precipitated proteins were analyzed by western blot. **H** RNA EMSAs to determine the domains of WDR5 responsible for the interaction with *AC142119.1* RNA. **P < 0.01

The above findings impelled us to explore the underlying mechanisms by which *AC142119.1* regulates the expression of *MYCN* in NB. Given that lncRNAs usually cooperated with specific proteins, such as chromatin-modifying complexes, transcriptional factors and RNP complexes, to conduct their regulatory roles [23]. Therefore, we performed RNA pull-down assay with *in*-*vitro*-transcribed biotinylated *AC142119.1* RNA to identify the protein partners of *AC142119.1* transcript in NB cells. The captured proteins were separated by SDS-PAGE and subjected to silver staining (Fig. 5A). Following mass spectrometry analysis identified 594 unique proteins were specifically enriched by *AC142119.1* RNA, rather than the antisense *AC142119.1* transcript. Among these proteins, WD repeat-containing protein 5 (WDR5) attracted our attention, which was a key component of histone H3K4 methyltransferase complex, and could catalyze H3K4me3 [24]. It is well-known that H3K4me3 is associated with the transcriptional activation of target genes [25]. Moreover, previous study demonstrated that WDR5 served as a tumor-promoter in NB and high level of WDR5 was positively correlated with poor prognosis of NB patients [26]. Therefore, we hypothesized that *AC142119.1* might cooperate with WDR5 to increase the H3K4me3 level and activate the transcription of *MYCN* gene in NB. We then validated the ability of WDR5 to bind with *AC142119.1* by western blot in the retrieved proteins from the RNA pull-down assay (Fig. 5B). Subsequent RIP assays performed with antibodies specific for WDR5 further consolidate the interaction between the endogenous *AC142119.1* transcript and WDR5 protein in SK-N-DZ and IMR-32 cells (Fig. 5C). We then explored the detailed interaction region between *AC142119.1* transcript and WDR5 protein. By using catRAPID (http://s.tartaglialab.com/page/catrapid_group), an algorithm to evaluate the binding propensity of protein-RNA pairs, we found that two fragments of *AC142119.1* RNA (1-400nt and 599-850nt) showed high affinity with WDR5 protein (Fig. 5D, E). Based on this prediction, biotin-tagged *AC142119.1* RNA truncations were constructed and used as probes for RNA pull-down assays (Fig. 5F). The results showed that both *AC142119.1*-Δ1 and *AC142119.1*-Δ2 could bind with WDR5 protein, whereas *AC142119.1*-Δ3 was unable to interact with WDR5, implying the 1-400 bp of *AC142119.1* transcript was critical for the interaction with WDR5 in NB cells (Fig. 5G). To further determine the region of WDR5 bound with *AC142119.1* RNA, the truncated forms of WDR5 (WDR5-D1, WDR5-D3, WDR5-D6 and WDR5-7) were also established and applied to RNA EMSA along with biotin-labeled *AC142119.1* probes in NB cells. We found that the domains of WDR5-D1 and WDR5-D7, rather than other domains, were indispensable for the interaction between WDR5 and *AC142119.1* RNA (Fig. 5H). Collectively, these findings proposed that *AC142119.1* could directly bind with WDR5 in NB cells.

### *AC142119.1 *recruits WDR5 protein to activate the transcription of *MYCN*

**Fig. 6:**
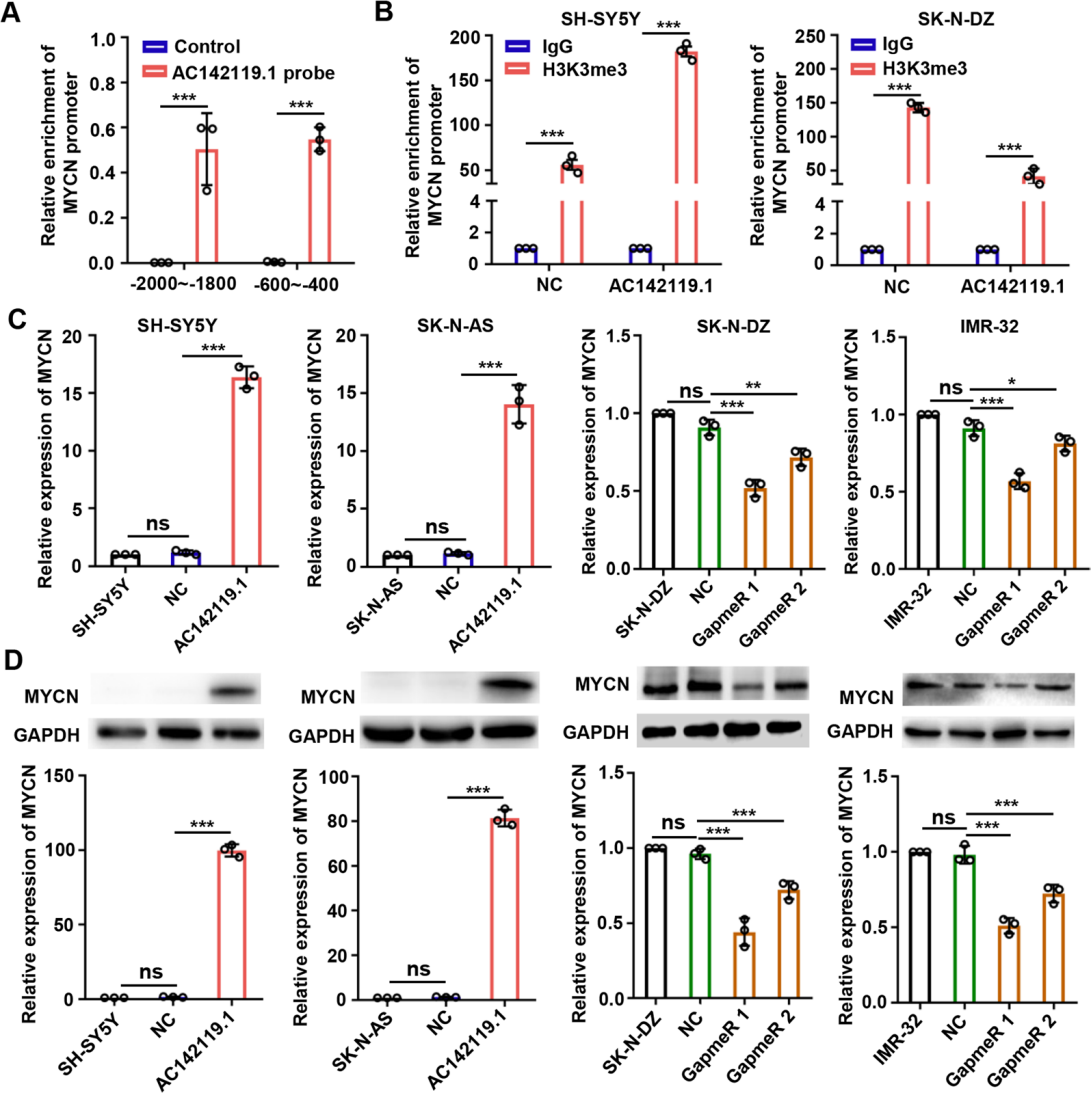
*AC142119.1* recruits WDR5 protein to activate the transcription of *MYCN*. **A** ChIRP assay was carried out to evaluate the enrichment of *AC142119.1* transcript on the promoter of *MYCN*. **B** ChIP assay was executed to detect the H3K4me3 modification of *MYCN* promoter. **C**, **D** The expression of mRNA (**C**) and protein (**D**) level of *MYCN* was assessed by qRT-PCR and western blot, respectively. *P < 0.05, **P < 0.01, ***P < 0.001, ns, no significance

To further validate our hypothesis, we performed ChIRP assay with biotin-labeled probes specific for *AC142119.1* in SK-N-DZ cells to observe the association between the *AC142119.1* transcript and *MYCN* promoter. As a result, we found that *AC142119.1* was significantly enriched at the regions of − 2000 – − 1800 bp and − 600 – − 400 bp from the transcription start site of *MYCN* promoter (Fig. 6A). In addition, the results of ChIP assays revealed that enforced expression of *AC142119.1* could markedly increase the histone active modification H3K4me3 of *MYCN* promoter while knockdown of *AC142119.1* generated an opposite effect (Fig. 6B). These data suggested that *AC142119.1* could recruit WDR5 to the promoter region of *MYCN* and enhance the H3K4me3 level of *MYCN* promoter. Next, we analyzed the effect of *AC142119.1* on the expression of *MYCN* in NB cells. Notably, both of the results of qRT-PCR and western blot assays showed that ectopic expression or depletion of *AC142119.1* resulted in significant increase or decrease of *MYCN* at both mRNA and protein levels, respectively (Fig. 6C, D). However, overexpression or knockdown of *MYCN* had no effect on *AC142119.1* expression levels in NB cells (Additional file 4: Fig. S3).

### *AC142119.1* promotes the progression of NB through interacting with WDR5

**Fig. 7:**
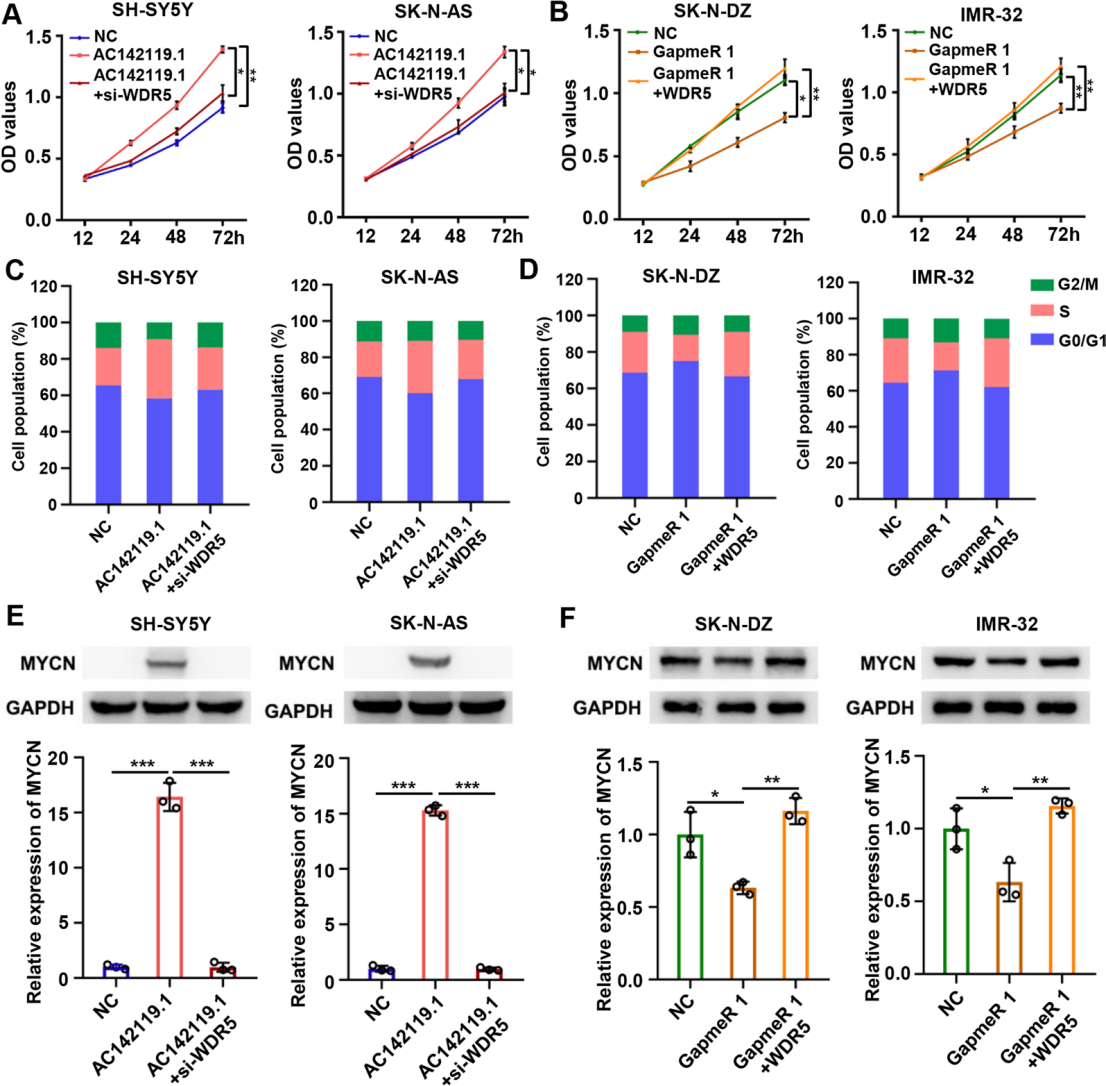
*AC142119.1* promotes the progression of NB through interacting with WDR5. **A**, **B** CCK-8 growth curves to detect the proliferation of NB cells co-transfected with indicated vectors, GapmerR or siRNA. **C**, **D** Cell cycle progression of NB cell lines were analyzed by flow cytometry.** E**, **F** Western blot assays to analyze the expression level of MYCN in NB cells. *P < 0.05, **P < 0.01, ***P < 0.001

**Fig. 8:**
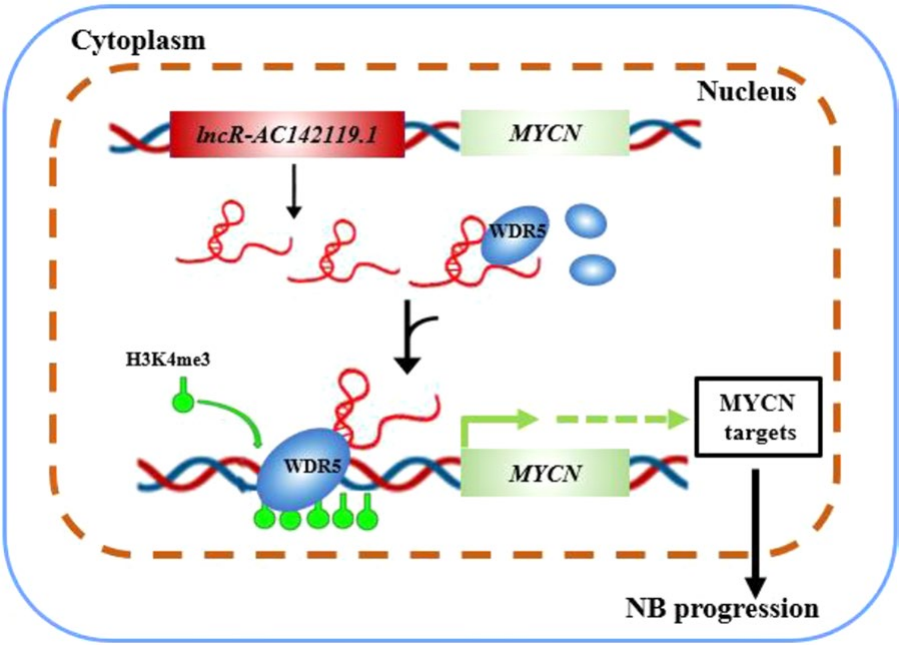
Schematic diagram shows *AC142119.1* activates the transcription of *MYCN through WDR5 protein*

To understand whether *AC142119.1* exerts its oncogenic role via WDR5 in NB, we conducted rescue experiments in NB cell lines. CCK-8 assays indicated that knockdown of WDR5 significantly abolished the increase of cell growth induced by overexpression of *AC142119.1* in SH-SY5Y and SK-N-AS cells (Fig. 7A), whereas ectopic expression of WDR5 could reverse the proliferation-suppressing effect produced by downregulation of *AC142119.1* in SK-N-DZ and IMR-32 cells (Fig. 7B). Consistently, cell cycle analyses showed that knockdown or overexpression of WDR5 abolished the promotive or inhibitory effects on cell cycle progression induced by upregulation or downregulation of *AC142119.1* in NB cells (Fig. 7C, D). Moreover, we also performed western blot analyses and found that the expression level of MYCN was upregulated or downregulated after ectopic expression or knockdown of *AC142119.1* in NB cells, which was rescued by silencing or overexpression of WDR5 (Fig. 7E, F). Altogether, the above findings suggested that *AC142119.1* recruited WDR5 protein to activate the transcription of *MYCN* and further promoted the progression of NB (Fig. 8).

## Discussion

Amplification of oncogene *MYCN* is considered as a major driver of NB and one of the strongest predictors of poor clinical outcomes of NB patients [9]. Although *MYCN* amplification or overexpression could be detected in approximately half of all high-risk NB cases, the molecular mechanism underlying the ectopic expression of *MYCN* has not been fully elucidated [27]. Recent years, lncRNAs have emerged as oncogenes or tumor suppressors in the initiation and progression of various human cancers, including NB [28–30]. However, whether individual lncRNAs contribute to the ectopic expression of *MYCN* in NB is largely unknown.

In the current study, by using microarray analysis, we firstly identified a novel *MYCN*-related lncRNA termed as *AC142119.1*, which was significantly elevated in *MYCN*-amplified NB cell lines and tissues, as well as NB tissues with advanced INSS stage and high risk. In addition, Pearson correlation analysis revealed a strong positive correlation between the expression of *AC142119.1* and *MYCN* mRNA in NB tissues. Importantly, NB patients with higher expression of *AC142119.1* had shorter overall survival time than those with low level of *AC142119.1*. By utilizing both loss- and gain-of-function approaches, we demonstrated that *AC142119.1* was an onco-lncRNA that could promote the proliferation and cell cycle progression of NB cell lines in vitro and accelerate tumor growth in vivo. Our findings uncovered the oncogenic role of *AC142119.1* in the progression of NB, supporting the possibility of *AC142119.1* as a promising predictor of malignant progression and potential therapeutic target for NB.

Amounting evidence demonstrated that lncRNAs could exert their regulatory role on neighboring protein-coding genes *in cis* by diverse mechanisms. For example, lncRNA transcript RBAT1 recruits hnRNPL to the promoter of oncogene *E2F3* to *ci*-activate its transcription, resulting in the tumorigenesis of retinoblastoma [31]. Moreover, lncRNA *TMPO-AS1* recruited FUS and p300 to the promoter of *TMPO* and formed a complex in situ, which activated the transcription of *TMPO* gene through increasing the H3K27ac level in its promoter [32]. In addition, lncRNA *RUNXOR* regulates the expression of *RUNX1* by altering the spatial structure of chromatin and enhancing histone H3K4me3 levels of *RUNX1* promoter [33]. In our study, to ascertain whether lncRNA could modulate the expression of *MYCN* in NB *in cis*, we sought out three differentially expressed lncRNA candidates (*AC142119.1*, *AC010145.3* and *AC130710.1*) located in the upstream or downstream of *MYCN* gene locus by bioinformatics analysis. Moreover, we found that none of them has been reported in cancers or other human diseases. *AC142119.1*, as the most upregulated one in *MYCN*-amplified NB cells, was then chosen for the further investigation. According to UCSC database, *AC142119.1* was predicted to produce two transcripts with length of 853nt and 642nt, respectively. Here, we presented a novel isoform of *AC142119.1* with a length of 924nt in SK-N-DZ cells. Moreover, we observed that *AC142119.1* transcript predominantly localized in the nucleus of NB cells and significantly correlated with the expression of *MYCN* mRNA in NB tissues. Our findings preliminarily testified that *AC142119.1* might work *in cis* to regulate the expression of *MYCN* in NB.

Our subsequent data of biotin-labeled RNA pull-down, RIP and RNA EMSA assays demonstrated that *AC142119.1* transcript could directly interact with WDR5. It has been well established that the highly-conserved nuclear protein WDR5 participates in the formation of multiple chromatin-modifying complexes, including SET1/MLL histone methyltransferase which catalyzes the trimethylation of histone H3K4 [34]. As a critical subunit of SET1/MLL complex, WDR5 promotes the assembly and activity of the complex by binding to the arginine-bearing motif of MLL [35]. SET1/MLL complexes mediated H3K4me3 is one of the most important histone modifications and usually selectively localizes to the promoter region and downstream transcription start sites to activate the transcription of target genes [36]. Previous study revealed that WDR5-H3K4me3 epigenetic axis could modulate the expression of *OPN* to enhance the immune escape of pancreatic cancer [37]. Additionally, WDR5 promotes the cell proliferation, self-renewal and chemoresistance of bladder cancer by activating multiple oncogenes via increasing H3K4me3 levels [38]. Under hypoxic conditions, WDR5 is upregulated and binds with histone deacetylase HDAC3, leading to the increased levels of histone H3K4me3 and overexpression of mesenchymal genes [39]. More importantly, in NB, WDR5 was positively associated with the expression of MYCN and formed a protein complex with MYCN to induce H3K4me3 and transcriptional activation of *MDM2* gene, thus resulting in the initiation and progression of NB [26]. However, WDR5 protein itself has no putative DNA-binding domains, and the mechanism of how WDR5 locates to target sites in chromatin is not certain.

Recent studies indicated that several lncRNAs, such as *TM4SF19-AS1*, *BLACAT2* and *SATB2-AS1* and *PTPRE-AS1*, could directly interact with WDR5 protein and serve as a guide to recruit chromatin regulatory complexes to target gene locus [40–43]. Consistent with the former studies, we proved that *AC142119.1* transcript could not only bind with WDR5 protein, but also directly interact with the promoter region of *MYCN*. More importantly, ChIP assays revealed that the H3K4me3 levels of *MYCN* promoter was significantly increased or decreased after overexpression or knockdown of *AC142119.1* in NB cells, meanwhile, the mRNA and protein levels of *MYCN* are correspondingly enhanced or inhibited. Importantly, manipulation of the expression of *MYCN* did not affect the expression level of *AC142119.1*, implying that *AC142119.1* is indeed an upstream regulator of *MYCN* gene. Our data suggests that *AC142119.1* activates the transcription of *MYCN* by recruiting WDR5 protein to the promoter of *MYCN* through the formation of a ternary complex with target DNA sequence, and increasing the H3K4me3 level of *MYCN* promoter in NB cells. However, further investigation was needed to fully understand the biological function and detailed mechanisms of *AC142119.1* in NB.

## Conclusion

In summary, our findings revealed that *AC142119.1* is an oncogenic lncRNA that is significantly elevated in NB tissues with *MYCN* amplification, advanced INSS stage or high risk. Moreover, *AC142119.1* serves as an epigenetic modifier to activate the transcription of *MYCN* gene by recruiting WDR5 protein to the promoter of *MYCN* and increasing its H3K4me3 levels, eventually leading to the malignant progression of NB. Therefore, our study provides new insights into the underlying mechanism of the ectopic expression of *MYCN* in NB and raises the possibility of *AC142119.1* to serve as a biomarker for the progression assessment and a therapeutic target for NB.

### Supplementary Information

**Additional file 1: Table S1. **The sequences of primers used in the current study. **Table S2. **The sequences of GapmeRs and siRNAs used in the current study.

**Additional file 2: Figure S1. **The identification of *AC142119.1* transcript.* A AC142119.1* transcript validated by RACE assays in SK-N-DZ cells. **B** The coding potential of AC142119.1 was assessed by PRIDE, PhyloCSF, CPAT, Bazzini small ORFs and Lee translation initiation sites, respectively.

**Additional file 3: Figure S2. **The expression level of Ki67 in xenograft tumors was determined by IHC staining. Scale bar, 100 μm.

**Additional file 4: Figure S3.** The expression of *AC142119.1* was detected by qRT-PCR after overexpression or knockdown of *MYCN* in NB cells. ns, no significance.

## Data Availability

The data supporting the results of the study were available from the corresponding author upon reasonable request.
