# Supplementary material for: LncRNA AC142119.1 facilitates the progression of neuroblastoma by epigenetically initiating the transcription of MYCN
Source: J Transl Med. 2023 Sep 23;21:659. doi: 10.1186/s12967-023-04535-3 (PMC10518117; doi:10.1186/s12967-023-04535-3)
Supplement: Supplementary file 1 — Additional file 1: Table S1. The sequences of primers used in the current study. Table S2. The sequences of GapmeRs and siRNAs used in the current study. [file 12967_2023_4535_MOESM1_ESM.docx]

**Table S1** The sequences of primers used in the current study.

| Identifier | Sequences |
| --- | --- |
| AC142119.1 | F: 5’-CTGTGCGGAGTCGAATTC-3’ |
|  | R: 5’-GCTTCCCAGGTTCAAGTGAT-3’ |
| MYCN | F: 5’-CGTTCCTCCTCCAACACCAA-3’ |
|  | R: 5’-CTTCTTCTGTGGGGGTGCAT-3’ |
| GAPDH | F: 5’-CAGCGACACCCACTCCTCCACCTT-3’ |
|  | R: 5’-ATGAGGTCCACCACCCTGTTGCT-3’ |
| 5' RACE | R1: 5’-GCAGAACTCGTTTCATTCAGGGCTTACA-3’ |
|  | R2: 5’-GGTGAAAGCATCAGGAATCTCCCA-3’ |
| 3' RACE | F1: 5’-GGAAACTGTAAGCCCTGAATGAAACGAGTT -3’ |
|  | F2: 5’-CAGCCAGGCGTGGTGGCTCACA-3’ |

**Table S2** The sequences of GapmeRs used in the current study.

| Difinition | Sequences |
| --- | --- |
| GapmeR 1 | 5’-TCTTACAGTGATGGAA-3’ |
| GapmeR 2 | 5’-TAAGCATCTTACATTG-3’ |
| si-NC | 5’-TTCTCCGAACGTGTCACGTAA-3’ |
| si-MYCN#1 | 5’‐CGGAGATGCTGCTTGAGAA‐3’ |
| si-MYCN#2 | 5’‐CAGCAGTTGCTAAAGAAAA‐3’ |
| si-WDR5 | 5’‐TCTGGAACCTTCAGACGAATT‐3’ |
