# Supplementary figures and images for: LncRNA AC142119.1 facilitates the progression of neuroblastoma by epigenetically initiating the transcription of MYCN
Source: J Transl Med. 2023 Sep 23;21:659. doi: 10.1186/s12967-023-04535-3 (PMC10518117; doi:10.1186/s12967-023-04535-3)

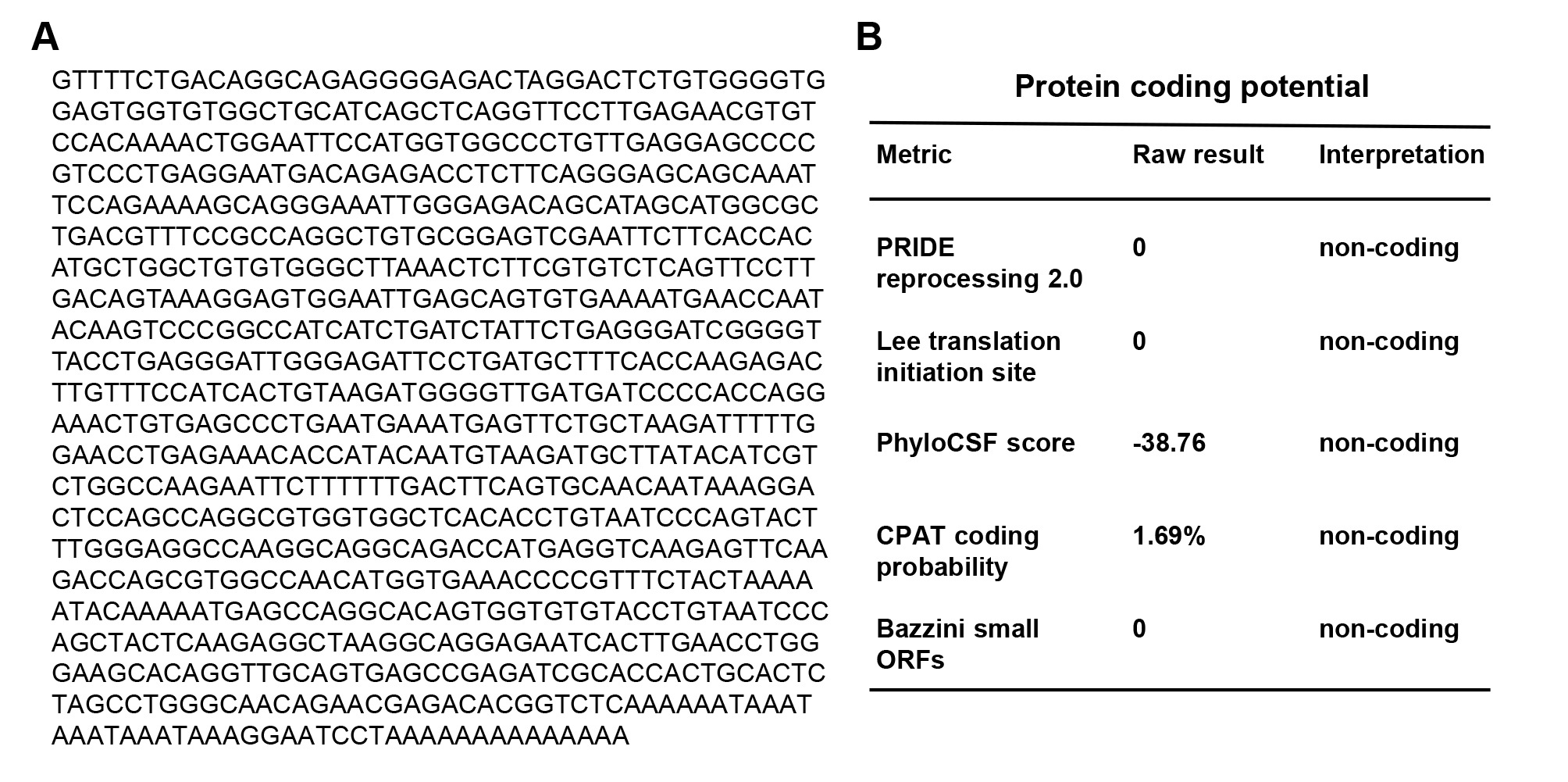

Supplement: Supplementary file 2 — Additional file 2: Figure S1. The identification of AC142119.1 transcript. A AC142119.1 transcript validated by RACE assays in SK-N-DZ cells. B The coding potential of AC142119.1 was assessed by PRIDE, PhyloCSF, CPAT, Bazzini small ORFs and Lee translation initiation sites, respectively. [file 12967_2023_4535_MOESM2_ESM.tif]

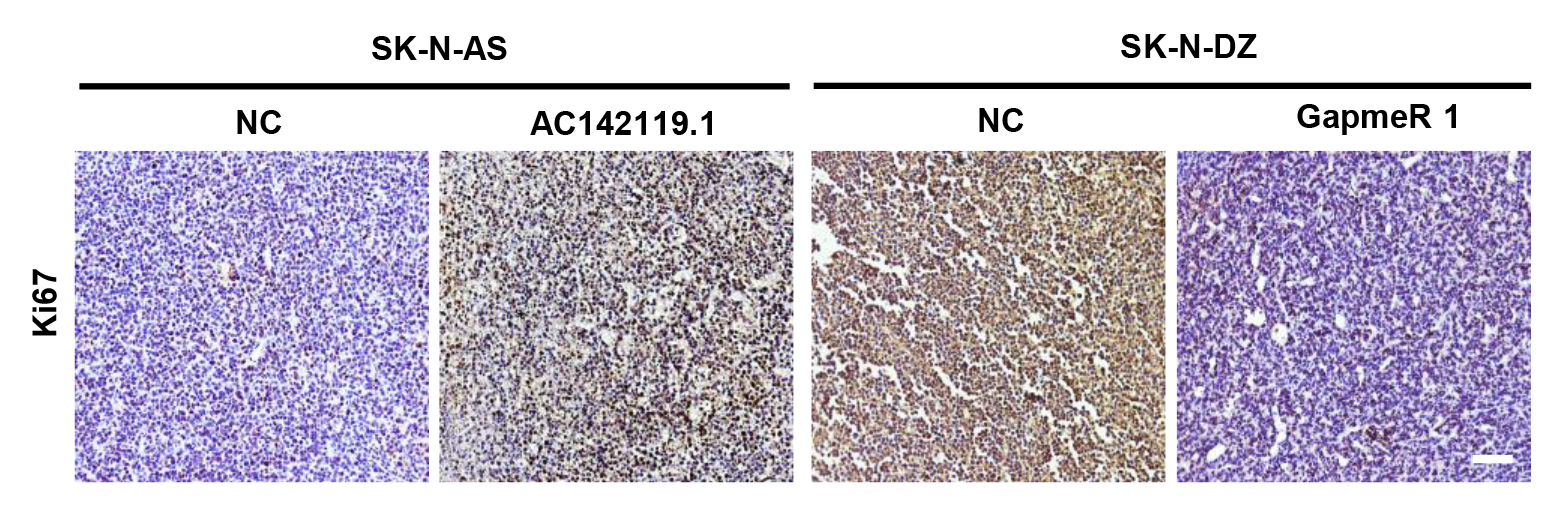

Supplement: Supplementary file 3 — Additional file 3: Figure S2. The expression level of Ki67 in xenograft tumors was determined by IHC staining. Scale bar, 100 μm. [file 12967_2023_4535_MOESM3_ESM.tif]

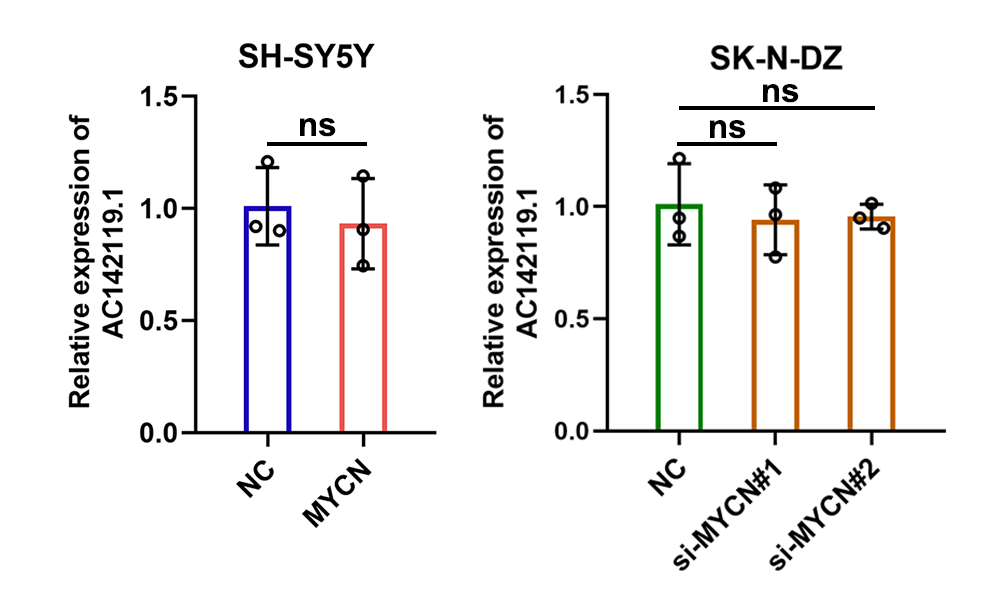

Supplement: Supplementary file 4 — Additional file 4: Figure S3. The expression of AC142119.1 was detected by qRT-PCR after overexpression or knockdown of MYCN in NB cells. ns, no significance. [file 12967_2023_4535_MOESM4_ESM.tif]
